# Pancreatic Drainage Into the Stomach After Pancreatic Resection

**DOI:** 10.1155/1991/35792

**Published:** 1991

**Authors:** Robert E. Hermann

**Affiliations:** The Cleveland Clinic Foundation One Clinic Center 9500 Euclid Avenue Cleveland Ohio 44195-5043 USA


16. Büchler, M., Malfertheiner, P., Schädlich, H., Nevalainen, T.J., Friess, H. and Beger, H.G. (1989) Role of Phospholipase A2 in human acute pancreatitis. *Gastroenterology*, **97**, 1521–1526
17. Gudgeon, A.M., Heath, D., Hurley, P., Shenkin, A., Jehanli, A., Patel, G., Wilson, C., Austen, B.M. and Imrie, C.W. (1988) Trypsinogen activation peptide (TAP) assay in severity assessment of acute pancreatitis. *Pancreas*, **3**, 598 (abstract)
18. Uhl, W., Büchler, M., Malfertheiner, P., Martini, M. and Beger, H.G. (1991) PMN-Elastase in comparison with CRP, antiproteas and LDH as indicators of necrosis in human acute pancreatitis. *Pancreas*, **6**, 253–259

Waldemar Uhl
Hans Günther Beger
Department of General Surgery
University of Ulm
Steinhövelstrasse 9
W-7900 Ulm
Germany

### PANCREATIC DRAINAGE INTO THE STOMACH AFTER PANCREATIC RESECTION

### ABSTRACT

*Delcore, R., Thomas, J.H., Pierce, G.E. and Hermreck, A.S. (1990) Pancreatogastrostomy: A safe drainage procedure after pancreatoduodenectomy. Surgery;* **108**: 641-647

**The purpose of this study was to evaluate the role of pancreaticogastrostomy as an alternative method of restoring pancreaticointestinal continuity after pancreatico-duodenectomy. Since 1975, 45 patients have undergone pancreaticogastrostomy after pancreaticoduodenectomy at our institution. Pancreaticoduodenectomy was performed for pancreatic carcinoma (24 patients), ampullary carcinoma (8 patients), duodenal carcinoma (4 patients), common bile duct carcinoma (4 patients), pancreatic islet cell carcinoma (1 patient), trauma (1 patient), extensive colon carcinoma (1 patient), chronic pancreatitis (1 patient), and gastroduodenal artery aneurysm (1 patient). There was one operative death, for an overall operative mortality rate of 2%, and seven patients had major postoperative complications, for an overall morbidity rate of 15%. No pancreatic anastomotic leaks or other complications related to the pancreaticogastrostomy occurred. Twenty-four patients have died of recurrent carcinoma, with a mean survival of 25 months (range, 5 to 66 months), and 20 patients are alive and well, with a mean follow-up of 27 months (range, 2 to 106 months). Eight of these patients are alive 2 or more years after operation and four do not have exocrine pancreatic insufficiency. This experience confirms that pancreaticogastrostomy is a safe method of pancreatic drainage after pancreaticoduodenectomy and suggests that it may have technical advantages and therefore merits more widespread application. (Surgery 1990; 108:641-7.)**

**PAPER DISCUSSION**

KEY WORDS: Pancreatogastrostomy, pancreatic resection

Since the introduction of pancreatoduodenectomy for the treatment of pancreatic and periampullary carcinoma, surgeons have been concerned about the best method to reconstruct the gastrointestinal tract with a specific concern about reestablishing pancreatic-intestinal continuity. Because of the frequency of problems with pancreatitis or leakage of the pancreatic anastomosis after pancreatojejunal reconstruction, some surgeons in the past have advocated oversewing the residual pancreas and ligating the pancreatic duct. Other surgeons have used the frequency of complications with pancreatic-intestinal re-anastomosis as an argument for total pancreatectomy. Various techniques of pancreatojejunostomy have been advocated including direct, mucosa to mucosa approximation of the pancreatic duct and jejunal mucosa, and insertion or "dunking"; of the entire pancreas into the jejunal lumen with a two layer anastomosis to provide a seal.

On many occasions, after resection of the head of the pancreas and the entire duodenum, there is tension on the anastomosis between the pancreas and jejunum. In such instances, surgeons have turned to other organs, such as the adjacent stomach, to reestablish continuity.

The authors trace the history of pancreatogastrostomy from its initial experimental use in canines by Tripodi and Sherwin in 1934, and its first clinical use by Waugh and Clagett, in 1946. Other, sporadic case reports have occurred subsequently, including an extensive use by surgeons at the University of Pennsylvania, Park, Mackie, and Rhoades, who reported its use in 28 patients.

The present series constitute 45 patients who have had pancreatogastrostomy performed after a Whipple operation with no pancreatic anastomotic leaks or other complications related to the pancreatogastrostomy. The authors have presented in this paper several theoretic and technical advantages which support the use of pancreatogastrostomy, including the anatomic proximity of the posterior gastric wall to the pancreas remnant, the security of suturing the pancreas into the stomach, and the postoperative decompression of the stomach which prevents pancreatic secretion buildup.

I have not used pancreatogastrostomy in my experience after pancreatoduodenectomy, since I have not had problems with pancreatojejunostomy as a method of reconstruction. This manuscript, as well as the reports of others, however, have made me look upon pancreatogastrostomy as a potentially safe operation which should be utilized by surgeons whenever they contemplate a difficult pancreatic-jejunal reanastomosis. It appears to be a safe alternative method for reconstruction of the pancreas back into the gastrointestinal tract after resection of the head of the pancreas.

Robert E. Hermann
The Cleveland Clinic Foundation
One Clinic Center
9500 Euclid Avenue
Cleveland, Ohio 44195-5043
United States of America

